# Characteristics and Treatment Outcomes for Patients of a Digital Psychology Service in Regional and Remote Parts of Australia

**DOI:** 10.1111/ajr.70032

**Published:** 2025-03-20

**Authors:** Lauren G. Staples, Blake F. Dear, Olav Nielssen, Nickolai Titov

**Affiliations:** ^1^ MindSpot Clinic, Macquarie University Sydney Australia

**Keywords:** anxiety, depression, digital mental health, regional, remote, rural, service implementation, service utilisation

## Abstract

**Objective:**

The MindSpot Clinic provides psychological assessment and treatment online or via the telephone to Australian residents. This study examines patient characteristics and treatment outcomes based on geographical location.

**Setting:**

MindSpot Clinic.

**Design:**

Retrospective analysis of patients who started an online assessment between January 2020 and December 2021 and provided a valid postcode that could be categorised as either Major City (*n* = 34 222) or Regional/Remote (*n* = 13 408).

**Participants:**

Adults residing in Australia and reporting symptoms of depression or anxiety.

**Main Outcome Measures:**

Demographic and satisfaction questionnaires, K‐10, PHQ‐9, GAD‐7.

**Results:**

Patient distribution was consistent with the national census, with 28% of patients residing in regional or remote locations. Comparison to patients from major cities showed that they were more likely to be residing in areas of high socioeconomic disadvantage. The regional/remote group included a higher proportion of females and a higher proportion of Indigenous patients. Despite baseline differences, online therapist‐guided treatment significantly decreased symptoms of anxiety and depression. Results were comparable to the major city group. For both groups, effect sizes were large (> 1.0 at post‐treatment), deterioration was low (< 3%) and reliable recovery rates were high (> 85%).

**Conclusion:**

Understanding differences and similarities based on geographic location is important for service provision. The MindSpot Clinic provides access to effective evidence‐based psychological care to patients across Australia, and the current results support the continued provision of digital psychology services in regional and remote areas of Australia.

**Summary**

- What is already known on this subject?
  ○ Geographical location can be a barrier to accessing mental health care. About 28% of the Australian population lives in regional and remote locations.
  ○ Digital psychology services have been successfully implemented in Australia, but few studies have specifically examined outcomes for patients located outside major cities, one of the main target groups of these services.
- What does this study add?
  ○ This study has the advantage of using a large sample of patients from across Australia who are accessing digital psychology services as part of routine care for symptoms of anxiety and depression.
  ○ This study is one of the few to directly compare mental health symptoms and treatment outcomes for patients residing in regional and remote Australia with patients located in or near major cities.
  ○ An important finding of the study is that digital psychological services provided to geographically disadvantaged patients are effective, acceptable, and able to reach a large group of patients who otherwise face barriers to psychological care.

## 1 Introduction

Depression and anxiety disorders are major contributors to the burden of disease globally [1] and in Australia [2]. However, even in countries with good systems of care, many people with these conditions do not receive evidence‐based psychological services [3]. Various barriers to care exist, including attitudinal barriers such as stigma or low perceived need, as well as structural barriers such as financial cost or geographical distance [4, 5]. Digital psychology services, which provide assessment and treatment via the internet or telephone, have been developed to help overcome those barriers, and now provide services to large numbers of patients in many countries as part of routine care [6, 7, 8, 9, 10, 11].

In Australia, the MindSpot Clinic provides digital psychology services online to adults with symptoms of anxiety and mood disorders. Previous studies have reported the acceptability and effectiveness of the service overall [9, 12, 13], and for specific subgroups defined by age [14, 15], medication use [16, 17] and cultural background [18]. However, none have specifically examined outcomes for patients located outside major cities.

In Australia, approximately 28% of the population lives in regional or remote areas [19]. This equates to about 7 million Australians, and evidence suggests that they have higher rates of mental and behavioural health conditions and higher levels of socioeconomic disadvantage [19]. The purpose of the current study is to provide data on service use and outcomes for patients in regional and remote locations compared with patients who live in the major cities. The specific aims of the study were: (1) to provide a descriptive analysis of demographics and service use for Australians residing in regional and remote locations and completing an online psychology assessment, and (2) to report treatment outcomes and patient satisfaction.

## 2 Methods

### 2.1 Study Design and Participants

**FIGURE 1:**
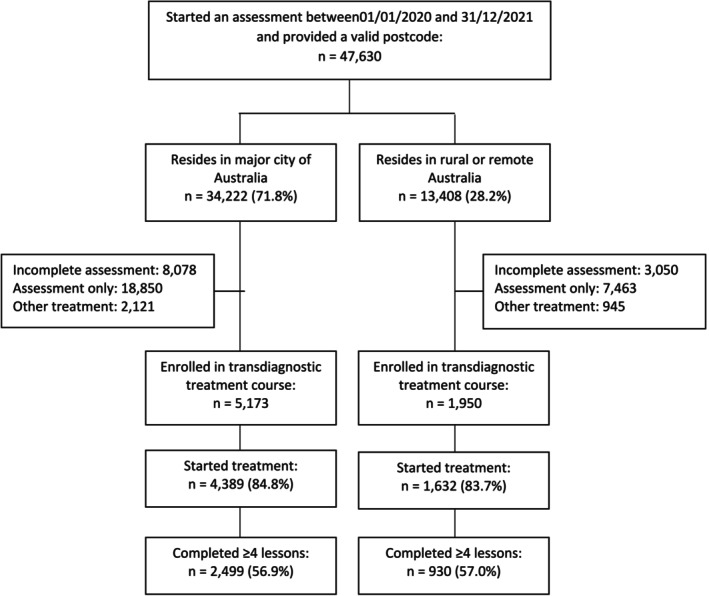
Patient Flow.

This study was designed as a retrospective uncontrolled observational cohort study. It included all people who started a MindSpot online assessment between 1st January 2020 and 31st December 2021 inclusive and provided a valid Australian residential postcode. MindSpot is a national digital psychology service that can be accessed directly via the website at no cost to consumers. All patients in the current study were aged 18 years or over if self‐referred or 16 years and over if directly referred by their general practitioner or other mental health professional. Ethical approval was provided by Macquarie University Human Research Ethics Committee (52022110184006). Patient flow is shown in Figure 1.

Residential postcodes were used to assign patients to a Remoteness Area as defined by the Australian Bureau of Statistics (ABS) [20]. Remoteness Areas divide Australia into five classes based on relative geographic access to services: Major Cities of Australia; Inner Regional Australia; Outer Regional Australia; Remote Australia; and Very Remote Australia. Postcodes were also used to assign patients to a decile on the Index of Relative Socioeconomic Disadvantage (IRSD) [21]. The IRSD summarises a range of information about the economic and social conditions of people and households within an area, with lower deciles indicating relatively greater disadvantage for that postcode.

### 2.2 Clinical Model

The MindSpot website provides information about symptoms and management of anxiety, depression and chronic pain, as well as information abouttreatment courses. People register with MindSpot via the website or telephone by completing a screening assessment. The assessment collects demographic, clinical and treatment history information and presents standardised symptom questionnaires. Patients are also asked about suicidal thoughts and plans. MindSpot operates under a robust framework of clinical governance that aligns with Australian national standards for mental health services and includes policies, systems, and protocols for identifying patients or others at risk. These are described in more detail elsewhere [22, 23].

People who start an assessment but do not complete it are sent information about managing symptoms and contact details for crisis services. Those who do complete the assessment are sent a report that provides information about the severity of their symptoms and how to access mental health services, including those offered by MindSpot. All patients who complete an assessment are encouraged to discuss their results via telephone with a therapist. People who complete an assessment can access a MindSpot digital treatment service unless a therapist considers them ineligible because their clinical presentation indicates the need for comprehensive or urgent face‐to‐face assessment. Those patients are then supported to access appropriate specialist services.

MindSpot (mindspot.org.au) offers a brief teletherapy service, a self‐directed online course, and eight therapist‐guided online treatment courses which have been developed and validated at the Macquarie University online research clinic (ecentreclinic.org). Four of the treatment courses are based on transdiagnostic principles aimed to teach psychological skills for managing symptoms of both anxiety and depression. In the current study, treatment outcomes were analysed for the subsample of patients who started one of the transdiagnostic courses (Wellbeing, Wellbeing Plus, Mood Mechanic, and Indigenous Wellbeing).

The courses have been validated and described in detail elsewhere [24, 25]. Briefly, the transdiagnostic Wellbeing courses consist of five lessons, delivered over 8 weeks. The first four lessons aim to teach CBT‐based skills, and the fifth lesson provides a summary and information for creating a relapse prevention plan. For analyses, the course is considered complete if the patient reads the four skills‐based lessons. In addition to the course materials, patients have access to regular support from a therapist. Patients are guided by the same therapist throughout the course via secure message or telephone contact. All therapists are mental health professionals, primarily registered psychologists, who have been trained in the delivery of digital psychological services.

### 2.3 Treatment Outcome Measures

Standardised symptom scales were administered to patients at assessment, week one of treatment (pre‐treatment), mid‐treatment, post‐treatment, and 3 months after course completion (3‐month follow‐up). For this study, outcomes on the Kessler Psychological Distress 10‐Item Scale (K‐10), Patient Health Questionnaire‐9 (PHQ‐9) and Generalised Anxiety Disorder 7‐Item Scale (GAD‐7) were analysed. The K‐10 consists of 10 items measuring non‐specific psychological distress [26]. Total scores range from 10 to 50, with scores ≥ 22 suggesting clinically significant levels of distress [27]. The PHQ‐9 consists of nine items and measures symptoms of major depressive disorder [28]. Total scores range from 0 to 27, and scores ≥ 10 indicate a likely diagnosis of depression. The GAD‐7 measures generalised anxiety disorder and is also sensitive to the presence of social phobia and panic disorder [29]. It consists of seven items, and total scores range from 0 to 21. Scores ≥ 8 indicate the probable presence of an anxiety disorder.

### 2.4 Statistical Analyses

At assessment, chi‐square analyses were used to test categorical group differences, and ANOVA was used to compare group differences on continuous variables. Bonferroni adjustment was used to account for multiple comparisons. Treatment outcomes were analysed using a modified intention‐to‐treatment protocol that included all patients who started a therapist‐guided transdiagnostic treatment course. Statistical significance was assessed using GEE models, with missing data imputed. Age was included as a covariate, given existing differences in age structures for the population groups under consideration [19]. An unstructured working correlation matrix was used, and a gamma distribution with a log link response scale was specified to address positive skewness in the dependent variable distributions. To assess clinical significance, Cohen's d effect sizes were calculated, and reliable change criteria were used to assess deterioration and improvement. Reliable change was calculated for patients completing the post‐treatment questionnaires. Reliable deterioration was defined as the proportion of patients showing an increase in scores from assessment to post‐treatment of at least one standard deviation (7 points on the K‐10, 6 points on the PHQ‐9, and 5 points on the GAD‐7), and reliable improvement was defined as the proportion of patients who showed a decrease in their scores of at least one standard deviation by post‐treatment. Patient satisfaction was also assessed at post‐treatment. Data were analysed using SPSS version 29.0.

## 3 Results

### 3.1 Sample Distribution

**FIGURE 2:**
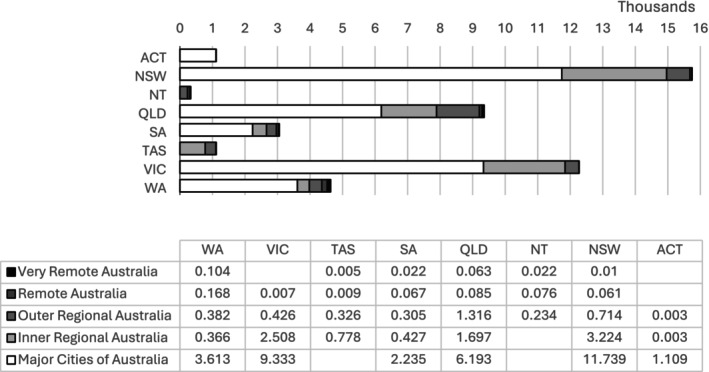
Distribution of patients by state and remoteness area.

Figure 2 shows the distribution of the study sample by state and remoteness area. For the purposes of further analyses, the regional and remote categories were collapsed into one group, and comparisons were conducted between the following groups: Major Cities (*n* = 34 222) and Regional/Remote (*n* = 13 408).

### 3.2 Patient Characteristics at Assessment

Table 1 compares demographics and symptoms at assessment for the whole sample. There were small but statistically significant differences between regional and remote patients for age and baseline symptom scores on the K‐10, PHQ‐9, and GAD‐7. However, these were not likely to be clinically significant, with Cohen's d effect sizes and confidence intervals (CIs) ≤ 0.16 (Age: *d* = 0.14, CI 0.12–0.16; K‐10: *d* = 0.04, CI 0.02 to 0.06, and both the PHQ‐9 and GAD‐7: *d* = 0.08, CI: 0.06–0.10). The regional and remote group included a higher proportion of females, a higher proportion who identified as Indigenous, and a higher proportion of patients born in Australia. They were also more likely to be taking psychotropic medication and more likely to have seen a mental health professional for their symptoms of anxiety or depression. Patients in the regional/remote group were more likely to be married and less likely to have a tertiary qualification. There were no differences in employment rate, reported thoughts or plans to self‐harm, or in reported days out of role due to symptoms.

**TABLE 1:** Comparison of demographics and symptoms at assessment.

	Major city (*n* = 34 222)		Regional/remote (*n* = 13 408)		Statistical significance
**Age**	$\bar{x}$	**SD**	$\bar{x}$	**SD**	
Mean age (SD) Age range: 18–90 years	33.9	(13.2)	35.8	(14.1)	*F* = 192.4, *p* < 0.001
**Gender**	***n***	**%**	***n***	**%**	
Male	8267	24.2%	2914	21.7%	*χ* ^2^ = 52.2, *p* < 0.001
Female	25 596	74.8%	10 409	77.6%	
Other	359	1.0%	85	0.6%	
**Cultural identity**					
Aboriginal or Torres Strait Islander	699	2.0%	743	5.5%	*χ* ^2^ = 1340.0, *p* < 0.001
Born in Australia: non‐Indigenous	22 924	67.0%	10 191	76.0%	
Born in country other than Australia	8317	24.3%	1475	11.0%	
No answer	2282	6.7%	999	7.5%	
**Education**					
Postgraduate degree	6211	18.1%	1712	12.8%	*χ* ^2^ = 863.7, *p* < 0.001
Undergraduate degree	8782	25.7%	2272	16.9%	
Other tertiary qualification	7507	21.9%	3918	29.2%	
Secondary or below	9265	27.1%	4495	33.5%	
No answer	2457	7.2%	1011	7.5%	
**Source of Income**					
Employed full or part‐time	19 273	56.3%	7640	57.0%	*χ* ^2^ = 1.7, *p* = 0.189
**Marital status**					
Married (registered and de facto)	11 093	32.4%	5058	37.7%	*χ* ^2^ = 121.2, *p* < 0.001
**Medication use**					
Current psychotropic medication	7331	21.4%	3654	27.3%	*χ* ^2^ = 198.8, *p* < 0.001
**Mental health professional use**					
Never seen mental health professional	11 823	34.5%	3703	27.6%	*χ* ^2^ = 251.5, *p* < 0.001
Previously but not currently	12 610	36.8%	5212	38.9%	
Currently seeing health professional	6477	18.9%	3144	23.4%	
No answer	3312	9.7%	1349	10.1%	
**Mean symptom scores at assessment**	$\bar{x}$	**SD**	$\bar{x}$	**SD**	
PHQ‐9	14.0	(6.2)	14.5	(6.2)	*F* = 61.2, *p* < 0.001
GAD‐7	11.8	(5.3)	12.2	(5.3)	*F* = 44.4, *p* < 0.001
K‐10	30.8	(7.6)	31.1	(7.6)	*F* = 17.5, *p* < 0.001
**Functional impact of symptoms**	$\bar{x}$	**SD**	$\bar{x}$	**SD**	
Whole days out of role in previous month	5.9	(7.6)	6.1	(7.9)	*F* = 2.7, *p* = 0.099
**Risk assessment**	***n***	**%**	***n***	**%**	
Low risk (no reported thoughts of self‐harm)	21 066	61.6%	8264	61.6%	*χ* ^2^ = 3.2, *p* = 0.352
Moderate (thought of self‐harm in past week)	6980	20.4%	2804	20.9%	
High (thoughts and plan for self‐harm)	773	2.3%	299	2.2%	
No answer	5403	15.8%	2041	15.2%	
**Relative socioeconomic disadvantage**					
1st decile (most disadvantaged)	1827	5.3%	1279	9.5%	*χ* ^2^ = 8178.7, *p* < 0.001
2nd decile	1476	4.3%	2046	15.3%	
3rd decile	1506	4.4%	1884	14.1%	
4th decile	2588	7.6%	2127	15.9%	
5th decile	2846	8.3%	2259	16.8%	
6th decile	3802	11.1%	1378	10.3%	
7th decile	3301	9.6%	882	6.6%	
8th decile	5193	15.2%	702	5.2%	
9th decile	5839	17.1%	644	4.8%	
10th decile (least disadvantaged)	5844	17.1%	207	1.5%	

*Note:* Mean ($\bar{x}$) and standard deviation (SD) are shown for continuous variables and number (*n*) and percentage (%) are shown for categorical variables.

**FIGURE 3:**
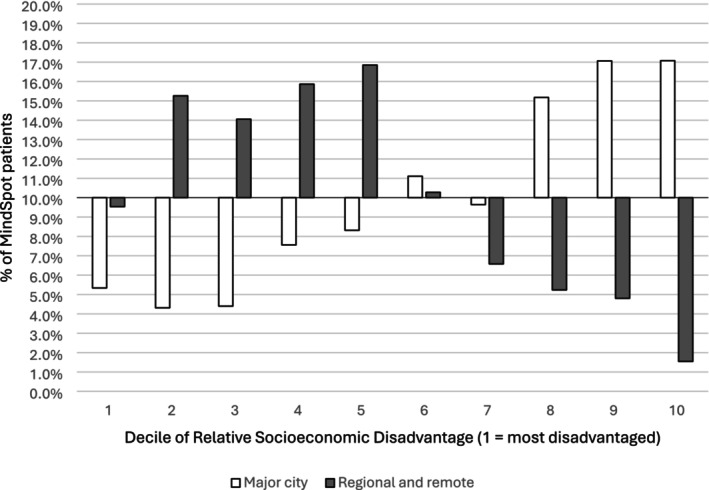
Relationship between location and relative socioeconomic disadvantage.

Patients in the regional/remote group were more likely to be residing in areas of greater socioeconomic disadvantage. As shown in Figure 3, regional/remote patients were over‐represented in the most disadvantaged deciles, with 72% residing in deciles 1 to 5, compared to just 30% of the major city group.

### 3.3 Treatment Outcomes

There was no difference between groups in the proportion of patients who started treatment: 84.8% (*n* = 4389) of enrolled patients in the major city group and 83.7% (*n* = 1632) of the enrolled patients in the regional/remote group started treatment (*p* > 0.05). There was also no difference in the proportions completing treatment (four or more lessons): 56.9% (*n* = 2499) of the major city group and 57.0% (*n* = 930) of the regional/remote group completed treatment. Demographic characteristics of the treatment group were similar to the demographic characteristics reported in Table 1 for the whole sample and are shown in Table S1.

**FIGURE 4:**
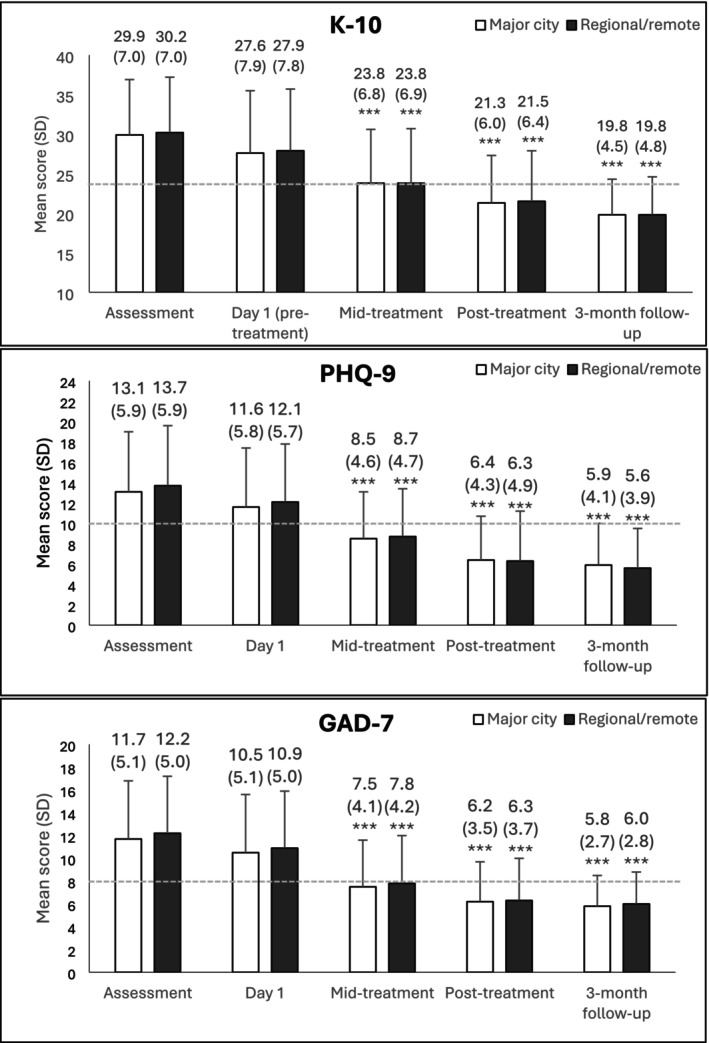
Estimated marginal means and standard deviations over time. Error bars represent standard deviation. Dotted lines represent the clinical cut‐off used for that measure. ***Significant compared to assessment at *p* < 0.001.

Group means over time on the K‐10, PHQ‐9 and GAD‐7 are shown in Figure 4. There were no significant between‐group differences or interaction effects (*ps* > 0.05). There were significant decreases over time for both groups on all symptom scales (K‐10: Wald's *χ* ^2^ = 11912.0, *p* < 0.001; PHQ‐9: Wald's *χ* ^2^ = 11311.3, *p* < 0.001; GAD‐7: Wald's *χ* ^2^ = 9669.6, *p* < 0.001). Mean scores on the PHQ‐9 and GAD‐7 were below clinical cut‐offs by mid‐treatment and remained under cut‐offs at post‐treatment and follow‐up for both groups.

The clinical significance of treatment is shown in Table 2. For both groups, effect sizes were large at post‐treatment and sustained at the 3‐month follow‐up. Rates of reliable deterioration were low for both groups (1.9%–2.6% for the major city group and 1.7%–2.9% for the regional/remote group), and reliable improvement rates were high (37.0%–41.2% and 38.7%–44.1% respectively).

**TABLE 2:** Clinical significance of treatment outcomes and patient satisfaction.

	K‐10		PHQ‐9		GAD‐7	
	Major city	Regional/Remote	Major city	Regional/Remote	Major city	Regional/Remote
Effect sizes from assessment to						
Day 1	0.31 [0.27–0.35]	0.31 [0.24–0.38]	0.26 [0.21–0.30]	0.28 [0.21–0.34]	0.24 [0.19–0.28]	0.26 [0.19–0.33]
Mid‐treatment	0.88 [0.84–0.93]	0.92 [0.85–0.99]	0.87 [0.83–0.91]	0.86 [0.86–1.01]	0.91 [0.86–0.95]	0.95 [0.88–1.03]
Post‐treatment	1.32 [1.27–1.37]	1.30 [1.22–1.37]	1.30 [1.25–1.34]	1.29 [1.29–1.44]	1.26 [1.21–1.30]	1.34 [1.27–1.42]
3‐month follow‐up	1.72 [1.67–1.77]	1.73 [1.65–1.81]	1.42 [1.37–1.46]	1.54 [1.54–1.70]	1.45 [1.40–1.49]	1.53 [1.45–1.61]
Reliable change at post‐treatment						
Deterioration	2.0% (49/2441)	2.2% (20/903)	1.9% (47/2432)	1.7% (15/897)	2.6% (64/2432)	2.9% (26/897)
Improvement	40.1% (979/2441)	40.9% (369/903)	37.0% (900/2432)	38.7% (347/897)	41.2% (1002/2432)	44.1% (396/897)

Satisfaction rates were also high. Of the patients that completed the satisfaction questionnaires at post‐treatment, 95% of both groups reported that they would recommend MindSpot to a friend (2269/2399 of the major city group and 840/882 of the regional/remote group), and 95% of both groups said that the course was worth their time (2275/2399 of the major city group and 842/882 of the regional/remote group).

## 4 Discussion

The current study describes patient characteristics and treatment outcomes for a large sample of patients from regional and remote Australia who accessed a digital psychology clinic for symptoms of anxiety and depression. A total of 13,408 patients residing outside of major cities were compared to patients residing in urban centres (*n* = 34 222). Results indicate that despite differences in sociodemographic characteristics, patients who enrolled in a therapist‐guided treatment course achieved large and significant reductions in symptoms following treatment regardless of location. These findings support the utility of digital psychology services for regional and remote Australians.

The distribution of this sample was strikingly similar to national data [19], with the regional/remote group accounting for 28% of the total. The regional and remote population was slightly older (mean age of 35.8 years compared to 33.9 years) and had a slightly higher proportion of female respondents (77.6% compared to 74.8%). Previous research has shown that barriers to care can be exacerbated by these factors. For example, older people living in rural areas have been identified as a specific group facing unmet mental health care needs [30, 31], while cost and geographical location present disproportional barriers for women located in rural areas of Australia accessing mental health services [32]. The current sample also included a higher proportion of Indigenous Australians in the regional and remote group (5.5%) compared to the urban group (2.0%). It is well known that there is a large disparity in health outcomes between Indigenous and non‐Indigenous Australians and an urgent need for culturally safe and effective mental health services [33]. We have previously published a comparison of Indigenous and non‐Indigenous users of MindSpot [34], which showed that MindSpot courses were effective in treating anxiety and depression in Indigenous Australians, and while our findings by no means close the gap, they do point to the importance of implementing culturally sensitive, evidence‐based digital psychology services that can be accessed by people across Australia. Patients residing outside of urban centres were also more likely to be living in areas of relative socioeconomic disadvantage. Almost three‐quarters of the regional and remote patients reported postcodes in the most disadvantaged deciles. Given that research indicates socioeconomic disadvantage is associated with increased psychological distress [35], our results support suggestions that more mental health resources need to be directed to disadvantaged areas, particularly those that are provided at no cost to consumers.

Interestingly, the regional and remote patients in this study reported higher levels of previous or current use of a mental health professional (62.3% compared to 55.7% of the urban group), and a higher rate of psychotropic medication use (27.3% compared to 21.4). Although speculative, digital services may be seen as a more accessible adjunct to primary treatment in areas where face‐to‐face allied services are limited. Differences may also be due to the small but statistically significant differences in demographic characteristics, but we acknowledge that regional/remote Australia is a heterogenous category and further subgroup analysis could be beneficial.

Despite the differences in demographic and clinical characteristics, treatment was effective for regional and remote patients, comparable to patients located in major cities. Patients both within and outside of major cities showed significantly improved symptoms of depression, anxiety and general distress by post‐treatment, and these improvements were maintained to 3‐month follow‐up. At post‐treatment, effect sizes were large for both groups and average symptom scores had declined to below clinical cut‐offs. Rates of deterioration were low (< 3%) and rates of improvement were high (> 37%). These results are consistent with previous outcomes reported by digital psychology services [6, 7, 8, 10, 11], and highlight that these services are effective and acceptable for regional and remote Australians.

The current study has some limitations. These include the use of a sample of treatment‐seeking adults who chose to engage with a digital service, which might not be generalisable to the entire population of people with anxiety and depression. We also note that the data were derived from patients accessing our services within the first 2 years of the COVID‐19 pandemic in Australia, when the burden of anxiety and depressive disorders increased [36], as did the use of telehealth and digital psychology services [37, 38]. The health consequences of the pandemic were unevenly distributed across demographic groups and geographical locations and were reported to have exacerbated existing socioeconomic differences [36, 39]. Replication of the current study or analysis of a multi‐year sample might reveal greater differences in outcomes.

Despite these limitations, a major strength of the current study is the very large and contemporary sample of patients from all parts of Australia. The results show the accessibility, real‐world effectiveness and acceptability of a digital psychology service specifically for patients located in geographically disadvantaged areas. Digital psychology services are not a replacement for face‐to‐face care in regional and remote regions, but are a complementary part of a supportive mental health framework. Such services can provide immediate and cost‐effective assessment and treatment, and also act as a triage service for patients seeking other forms of support. The successful implementation of digital services in routine care is challenging and requires clear processes and governance, but they can be a particularly valuable means of addressing barriers to mental health care and engaging hard to reach populations.

## Author Contributions

**Lauren G. Staples:** conceptualization, methodology, formal analysis, data curation, writing – original draft. **Blake F. Dear:** writing – review and editing. **Olav Nielssen:** writing – review and editing. **Nickolai Titov:** writing – review and editing, conceptualization.

## Ethics Statement

This study was approved by the Human Research Ethics Committee at Macquarie University (52022110184006).

## Supporting information


Table S1.

## Data Availability

The data that support the findings of this study are available on request from the corresponding author. The data are not publicly available due to privacy or ethical restrictions.
