# Supplementary material for: Characteristics and Treatment Outcomes for Patients of a Digital Psychology Service in Regional and Remote Parts of Australia
Source: Aust J Rural Health. 2025 Mar 20;33(2):e70032. doi: 10.1111/ajr.70032 (PMC11924299; doi:10.1111/ajr.70032)
Supplement: Supplementary file 1 — Table S1. [file AJR-33-0-s001.docx]

S*upplement Table S1. Comparison of demographics and symptoms for patients who started treatment*

|  | Major City  (n = 4,389) | | Regional/Remote  (n = 1,632) | | Significance |
| --- | --- | --- | --- | --- | --- |
| **Age** | ***x̄*** | **SD** | ***x̄*** | **SD** |  |
| Mean age (SD)  Age range: 18 – 90 years | 37.8 | (13.6) | 39.9 | (14.3) | F = 26.3, *p* < .001 |
| **Gender** | **n** | **%** | **n** | **%** |  |
| Male | 1311 | 29.9% | 399 | 24.4% | χ^2^ = 18.1,  *p* < .001 |
| Female | 3058 | 69.7% | 1228 | 75.2% |  |
| Other | 20 | 0.5% | 5 | 0.3% |  |
| **Cultural identity** | **n** | **%** | **n** | **%** |  |
| Aboriginal or Torres Strait Islander | 55 | 1.3% | 49 | 3.0% | χ^2^ = 154.2,  *p* < .001 |
| Born in Australia: non-Indigenous | 2975 | 67.8% | 1317 | 80.7% |  |
| Born in country other than Australia | 1316 | 30.0% | 244 | 15.0% |  |
| No answer | 43 | 1.0% | 22 | 1.3% |  |
| **Education** | **n** | **%** | **n** | **%** |  |
| Postgraduate degree | 1138 | 25.9% | 337 | 20.6% | χ^2^ = 122.4,  *p* < .001 |
| Undergraduate degree | 1428 | 32.5% | 363 | 22.2% |  |
| Other tertiary qualification | 1031 | 23.5% | 515 | 31.6% |  |
| Secondary or below | 726 | 16.5% | 393 | 24.1% |  |
| No answer | 66 | 1.5% | 24 | 1.5% |  |
| **Source of Income** | **n** | **%** | **n** | **%** |  |
| Employed full or part-time | 2907 | 66.9% | 1072 | 66.2% | χ^2^ = .3, *p* = .578 |
| **Marital status** | **n** | **%** | **n** | **%** |  |
| Married (registered and de facto) | 1847 | 42.7% | 827 | 51.3% | χ^2^ = 35.5, *p* < .001 |
| **Medication use** | **n** | **%** | **n** | **%** |  |
| Current psychotropic medication | 1278 | 29.2% | 634 | 39.0% | χ^2^ = 52.5, *p* < .001 |
| **Mental health professional use** | **n** | **%** | **n** | **%** |  |
| Never seen mental health professional | 1120 | 25.5% | 296 | 18.1% | χ^2^ = 65.9,  *p* < .001 |
| Previously but not currently | 2075 | 47.3% | 735 | 45.0% |  |
| Currently seeing health professional | 1186 | 27.0% | 596 | 36.5% |  |
| No answer | 8 | 0.2% | 5 | 0.3% |  |
| **Symptom scores at assessment (mean & SD)** | ***x̄*** | **SD** | ***x̄*** | **SD** |  |
| PHQ-9 | 13.1 | (5.9) | 13.7 | (5.9) | F = 12.2, *p* < .001 |
| GAD-7 | 11.7 | (5.1) | 12.2 | (5.0) | F = 12.7, *p* < .001 |
| K-10 | 29.9 | (7.0) | 30.2 | (7.0) | F = 2.1, *p* = .140 |
| **Functional impact of symptoms (mean & SD)** | ***x̄*** | **SD** | ***x̄*** | **SD** |  |
| Whole days out of role in previous month | 5.3 | (7.2) | 5.5 | (7.4) | F = 1.3, *p* = .246 |
| **Risk assessment** | **n** | **%** | **n** | **%** |  |
| Low risk (no reported thoughts of self-harm) | 3342 | 76.4% | 1252 | 76.8% | χ^2^ = .2,  *p* = .924 |
| Moderate (thought of self-harm in past week) | 958 | 21.9% | 353 | 21.6% |  |
| High (thoughts and plan for self-harm) | 75 | 1.7% | 26 | 1.6% |  |
| No answer | 0 | 0 | 0 | 0 |  |
| **Relative socioeconomic disadvantage** | **n** | **%** | **n** | **%** |  |
| 1^st^ decile (most disadvantaged) | 178 | 4.1% | 144 | 8.8% | χ^2^ = 1118.3,  *p* < .001 |
| 2^nd^ decile | 177 | 4.0% | 240 | 14.7% |  |
| 3^rd^ decile | 190 | 4.3% | 240 | 14.7% |  |
| 4^th^ decile | 281 | 6.4% | 242 | 14.8% |  |
| 5^th^ decile | 340 | 7.7% | 285 | 17.5% |  |
| 6^th^ decile | 480 | 10.9% | 158 | 9.7% |  |
| 7^th^ decile | 407 | 9.3% | 115 | 7.0% |  |
| 8^th^ decile | 725 | 16.5% | 102 | 6.3% |  |
| 9^th^ decile | 787 | 17.9% | 77 | 4.7% |  |
| 10^th^ decile (least disadvantaged) | 824 | 18.8% | 29 | 1.8% |  |

Mean (***x̄)*** and standard deviation (SD) are shown for continuous variables and number (n) and percentage (%) are shown for categorical variables.
